# Normal cognition in transgenic BRI2-Aβ mice

**DOI:** 10.1186/1750-1326-8-15

**Published:** 2013-05-12

**Authors:** Jungsu Kim, Paramita Chakrabarty, Amanda Hanna, Amelia March, Dennis W Dickson, David R Borchelt, Todd Golde, Christopher Janus

**Affiliations:** 1 Department of Neuroscience and Center for Translational Research in Neurodegenerative Disease, University of Florida, 1275 Center Dr., Box 100159, Gainesville, FL 32610, USA; 2 Department of Neuroscience, Mayo Clinic, Jacksonville, FL 32224, USA; 3 Current address: Department of Neurology, Washington University School of Medicine, 660 S. Euclid Box 811, St. Louis, MO 63110, USA

**Keywords:** Alzheimer’s disease, Mouse models, Amyloid-β, Amyloid plaques, Cognition

## Abstract

**Background:**

Recent research in Alzheimer’s disease (AD) field has been focused on the potential role of the amyloid-β protein that is derived from the transmembrane amyloid precursor protein (APP) in directly mediating cognitive impairment in AD. Transgenic mouse models overexpressing APP develop robust AD-like amyloid pathology in the brain and show various levels of cognitive decline. In the present study, we examined the cognition of the BRI2-Aβ transgenic mouse model in which secreted extracellular Aβ1-40, Aβ1-42 or both Aβ1-40/Aβ1-42 peptides are generated from the BRI-Aβ fusion proteins encoded by the transgenes. BRI2-Aβ mice produce high levels of Aβ peptides and BRI2-Aβ1-42 mice develop amyloid pathology that is similar to the pathology observed in mutant human APP transgenic models.

**Results:**

Using established behavioral tests that reveal deficits in APP transgenic models, BRI2-Aβ1-42 mice showed completely intact cognitive performance at ages both pre and post amyloid plaque formation. BRI2-Aβ mice producing Aβ1-40 or both peptides were also cognitively intact.

**Conclusions:**

These data indicate that high levels of Aβ1-40 or Aβ1-42, or both produced in the absence of APP overexpression do not reproduce memory deficits observed in APP transgenic mouse models. This outcome is supportive of recent data suggesting that APP processing derivatives or the overexpression of full length APP may contribute to cognitive decline in APP transgenic mouse models. Alternatively, Aβ aggregates may impact cognition by a mechanism that is not fully recapitulated in these BRI2-Aβ mouse models.

## Background

Mouse models overexpressing *APP* and/or presenilin1 (*PSEN1*) genes implicated in familial AD (FAD) are powerful tools to study cerebral Aβ accumulation and its effect on cognition [1]. Though many APP transgenic mouse models have been shown to develop relevant AD-related Aβ pathology and exhibit cognitive impairment within 6 to 12 months of age [2-4], the attempts to find specific correlations between molecular markers of Aβ processing and cognitive deficits in these mice, which express high levels of APP, creates challenges in deciphering the basis for cognitive changes that may occur in a model.

The overexpression of full-length human APP in transgenic mice generates multiple biologically active APP proteolytic fragments, potentially capable of altering behaviour. For example, the accumulation of amino-terminal-soluble APPβ (sAPPβ) and/or β-carboxyl-terminal fragments (β-CTF) may affect long-term potentiation (LTP) [5] and memory acquisition in mouse models [6]. Thus, the relative contribution of Aβ and/or other APP metabolites to cognitive deficits in the APP over-expression models is challenging to resolve. Indeed, the roles of APP and/or Aβ in mediating cognition do not have to be mutually exclusive, as complex synergistic interactions may occur. In this study, we attempted to elucidate whether selective overexpression of Aβ via a BRI2 fusion strategy that results in efficient Aβ secretion, and in the case of BRI2Aβ1-42 CNS amyloid deposition, produces cognitive deficits. The BRI2 transgnic mice studied express Aβ1-40, Aβ1-42, or both Aβ1-40/Aβ1-42 peptides in the secretory pathway utilizing an engineered *BRI2* gene in which a natural sequence encoding the 23-amino-acid amyloid Abri peptide at the C-terminus of the type II trans-membrane BRI protein was replaced with a sequence encoding either Aβ1-40 or Aβ1-42 [7]. The Abri peptide is naturally cleaved by proprotein convertases [8], and the Aβ peptides are released by proprotein convertase cleavage in the late secretory compartment. Both BRI2-Aβ1-40 and BRI2-Aβ1-42 mice show the presence of the respective soluble Aβ peptides at the age of 3 months at levels ~2- to 3-fold higher than the levels of Aβ in 3- to 6-month-old APP Tg2576 mice. However, only BRI2-Aβ1-42 mice develop Aβ amyloid pathology in the brain at the age of 12 months with robust compact Aβ plaques in the hippocampus at 14 months [7]. Given the reports of cognitive deficits in mice that express mutant APP and the association, in some cases, of these deficits with amyloid deposition, we sought to determine whether the BRI2-Aβ1-42 mice manifest detectable cognitive deficits. We evaluated all BRI2-Aβ mice in a battery of non-mnemonic as well as mnemonic tests, used previously in our lab to characterize phenotypes of PS1 and APP transgenic mice [9-13]. Specifically, to investigate non-mnemonic behavior, we focused on the exploration and emotional behavior (open-field test [14,15]), on motor coordination and balance (rotarod test [16,17]), and on swimming ability and the orientation to a visible cue (visible platform water maze test [10,11]). Cognitive evaluation encompassed (i) conditioned context and tone fear memory evaluated in FC test, a form of Pavlovian associative learning that employs pairing of an initially neutral explicit cue as a conditional stimulus (CS), such as tone, with an aversive unconditional stimulus (US) (e.g. foot shock) [18,19]. The memory of the context in which an animal experienced US depends on intact hippocampus [20], while the association between the tone stimulus and US depends on amygdala [19]. (ii) Spatial reference memory version of the WM test which depends on intact hippocampus [21,22], and (iii) conditioned taste aversion, an implicit associative learning, a form of Pavlovian classical conditioning, which is relatively independent of motor behavior, implicates several brain structures (e.g. parabrachial nucleus, amydgala, insular cortex), cholinergic system and NMDA receptors [23].

Here we report that BRI2-Aβ mice, including the BRI2-Aβ1-42 mice show a surprising lack of cognitive impairment; a finding that may have implications regarding the mechanisms by which mice overexpressing mutant APP develop cognitive deficits.

## Results

### Amyloid-β brain pathology in BRI2-Aβ mice

**Figure 1:**
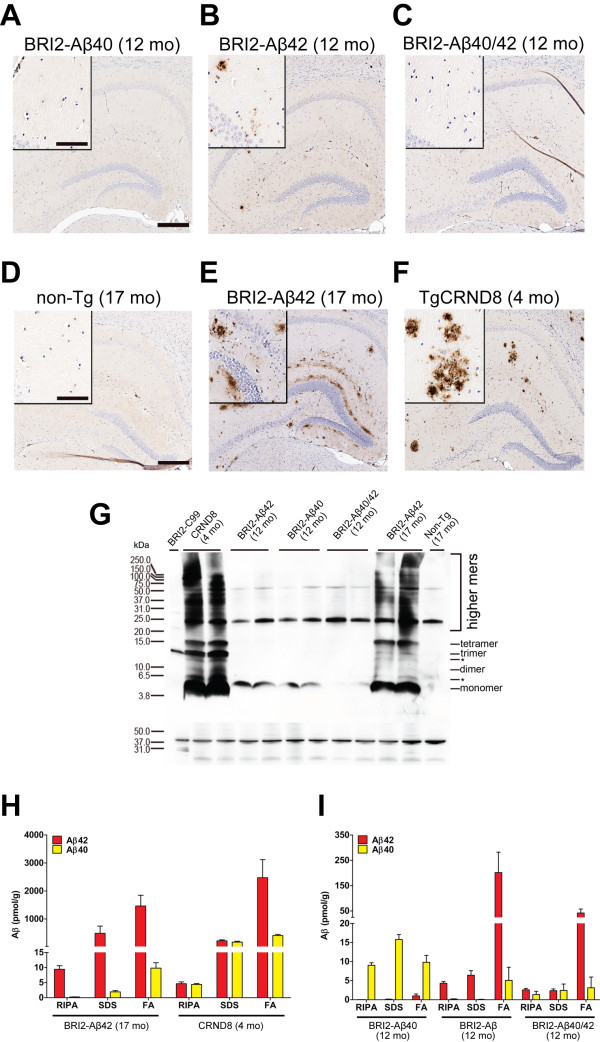
**Aβ pathology in BRI2-Aβ transgenic mice.** Images of the hippocampal region immunostained with an anti-Aβ1-16 antibody (**A-C**) 12-month-old BRI2-Aβ, (**D**) 17-month-old non-Tg, (**E**) 17-month-old BRI2-Aβ1-42 and (**F**) 4-month-old APP CRND8 mice. (**G**) Representative 82E1 immunoblots of 4-month-old CRND8 mice and 17-month-old BRI2-Aβ1-42 mice show increased presence of Aβ oligomers (di/tri/tetrameric and higher molecular weight species) as compared to younger 12-month-old BRI2-Aβ and 17-month-old non-Tg mice. The first, control lane includes H4 cell lysate expressing BRI2-C99 fusion protein. A band between 10 and 15 kDa represents C99 peptide. Molecular weight markers are indicated on the left (kD). The lower panel represents the 82E1 blot re-probed with anti-β-actin antibody to depict loading amount (* Bands not characterized). (**H**-**I**) RIPA soluble and insoluble Aβ levels in the brains of BRI2-Aβ and CRND8 mice were measured by Aβ ELISA after sequential extraction using RIPA, SDS, and FA (n = 10-15 per genotype). Scale bars (**A**, **D**): 250μm, inserts-80μm. Error bars represent s.e.m. For brevity and clarity of presentation, we used shorter labels of BRI2-Aβ lines (BRI2-Aβ40; BRI2-Aβ42; BRI2-Aβ40/42) in the panels of all figures.

Post-mortem analysis of Aβ histopathology and Aβ levels analyzed biochemically in the brains of BRI2-Aβ mice is depicted (Figure 1). As previously reported, BRI2-Aβ1-40 mice did not develop amyloid deposits (Figure 1A), whereas aged BRI2-Aβ1-42 mice developed Aβ1-42 plaques in the cortex and hippocampus (Figure 1B) confirming our previous results [7,24]. In 17-month-old BRI2-Aβ1-42 mice, amyloid plaque pathology as well as RIPA soluble and insoluble Aβ levels were comparable to levels found in APP CRND8 mice at the age of 4 months (Figure 1E-F, and H), when CRND8 mice showed reliable cognitive impairment in our previous studies [9,13]. Biochemical analysis of Aβ revealed the presence of higher molecular weight Aβ oligomeric species in brain tissue of BRI2-Aβ1-42 mice (Figure 1G). Bitransgenic BRI2-Aβ1-40/BRI2-Aβ1-42 mice showed reduced Aβ, but not absent, deposition (Figure 1C and I), confirming our previous study [24], which demonstrated the anti-amyloidogenic propensity of Aβ1-40 with respect to at least early stage amyloid deposition. The levels of RIPA-soluble and insoluble, formic acid extractable, Aβ1-42 were significantly correlated (r_*s*_ = 0.99, *p* < 0.001), and the combined total pool of biochemically evaluated Aβ1-42 was also significantly correlated with Aβ burden pathology (*r*_*s*_ = 0.97, *p* < 0.001) in 17-mo-old BRI2-Aβ1-42 mice, as seen previously in APP CRND8 mice [10]. The comparison of biochemically extracted Aβ between males and females in each of the BRI2-Aβ line did not reveal significant differences in Aβ levels (data not shown).

### BRI2-Aβ mice show uncompromised conditioned fear memory at the early stage of Aβ deposition

**Figure 2:**
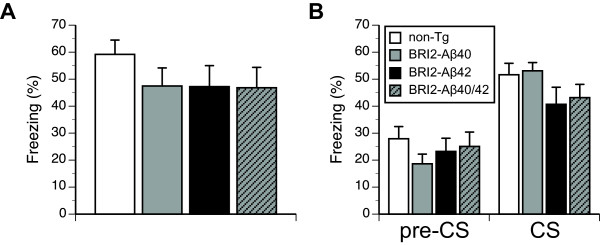
**Conditioned fear memory of BRI2-Aβ mice at the onset (12 months of age) of Aβ1-42 deposition in BRI2-Aβ1-42 mice.** (**A**) Context fear memory and (**B**) tone fear memory tested after 48h and 72h following CS-US pairing. Freezing to tone (CS) was stronger than freezing to altered testing chamber (pre-CS) phase (*p* < 0.01, phase effect). Error bars represent s.e.m.

We initially evaluated all BRI2-Aβ lines and non-transgenic (non-Tg) littermates at 12 months of age (Study 1) when BRI2-Aβ1-42 mice show the onset of amyloid pathology defined as deposition of Aβ1-42 in plaques in the hippocampus [7]. We employed delay FC paradigm, which proved to be sensitive paradigm used previously to detect progressive memory decline in APP CRND8 mice [25], and to demonstrate memory decline in Tg2576 mice at ages preceding Aβ aggregation and plaque deposition [26]. The comparison between non-transgenic (non-Tg) and the three BRI2-Aβ (BRI2-Aβ1-40, BRI2-Aβ1-42, and BRI2Aβ1-40/Aβ1-42) lines did not reveal significant genotype differences in the initial exploration of the training chamber (Table 1) or in freezing rates during contextual and tone memory tests (Figure 2, Table 1). Males and females showed comparable context and tone memories, however, males froze longer than females during the pre-CS phase of the tone test (F(1,64) = 4.8, p < 0.05), indicating their increased generalization of the training context. There was no significant interaction between the genotype and sex in any of these analyses, indicating that sex did not influence the fear conditioned memory within studied genotypes. In conclusion, the results of Study 1 demonstrated an uncompromised hippocampus-dependent contextual fear memory, and amygdala-dependent tone fear memory in all BRI2-Aβ lines tested at the early stages of Aβ1-42 pathology in BRI2-Aβ1-42 mice. Additionally, our analyses revealed that the contextual and tone fear memory scores were not affected by sex of mice within each genotype and were not significantly correlated with the levels of biochemically extracted Aβ in each of the studies BRI2-Aβ line (data not shown).

**Table 1:** The analysis of the genotype effect in behavioral tests

	**Test/behavior**	**Age**	**F**	**df**_**tr**_**,df**_**er**_	**sig**	**ω**^**2**^
Study 1	FC/exploration	12mo	0.9	3,64	0.44	0.00
	FC/context memory		1.0	3,64	0.41	0.00
	FC/pre-CS		0.7	3,64	0.53	0.00
	FC/CS tone memory		1.7	3,64	0.18	0.02
Study 2	OF/anxiety index	14.2mo	0.7	2,21	0.51	0.00
	OF/latency to explore		0.3	2,21	0.92	0.00
	OF/path length		1.3	2,21	0.30	0.00
	OF/path tortuosity		1.7	2,21	0.20	0.00
	OF/object exploration		1.2	2.21	0.82	0.00
	RR/latency to fall	14.6mo	2.1	2,20	0.19	0.00
	VPWM/path	15.1mo	0.9	2,21	0.42	0.00
	VPWM/speed		0.4	2,21	0.79	0.00
	VPWM/path tortuosity		2.3	2,21	0.12	0.09
	SRWM/search path	15.3mo	0.1	2,21	0.95	0.00
	SRWM/swim speed		2.6	2,21	0.10	0.06
	SRWM/%path in TQ		0.2	2,21	0.81	0.00
	*SRWM/search path	*17mo	0.4	1,19	0.53	0.00
	*SRWM/swim speed		0.4	1,19	0.52	0.00
	*SRWM/%path in TQ		0.3	1,19	0.86	0.00
	CTA/D2	15.8	1.0	2,20	0.40	0.00
	CTA/D10-D15		1.0	2,20	0.40	0.03

The ANOVA results pertain only to the differences between non-Tg, BRI2-Aβ1-40, BRI2-Aβ1-42, and BRI2-Aβ1-40/Aβ1-42 mice in Study 1, and the differences between non-Tg, BRI2-Aβ1-40, BRI2-Aβ1-42 mice in Study 2. Other significant main effects of sex, repeated testing, and their interactions, as well as comparisons against chance performance are reported in the text. In Study 1 12mo-old mice were tested in a fear conditioning (FC) test. In Study 2 the mice were tested in a battery of tests including open-field (OF), rotarod (RR), visible (VP) and spatial reference (SR) memory of the water maze (WM) tests, and conditioned taste aversion (CTA) tests following longitudinal experimental design. Results indicated by * pertain to the SRWM test carried out on a cohorts of naïve 17 mo-old non-Tg and BRI2-Aβ1-42 mice. ω^2^ represents the effect size due to the genotype of mice.

### Cognitive behavior of BRI2-Aβ mice was not compromised at the stage of overt Aβ deposition into plaques

**Figure 3:**
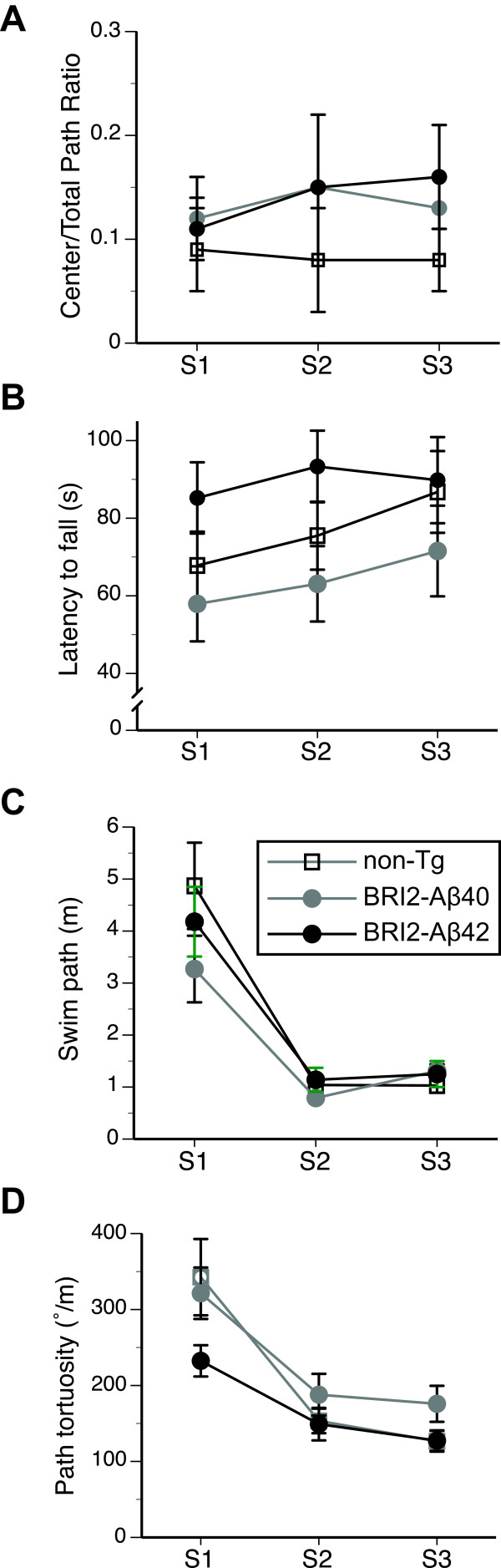
**Exploratory activity and motor coordination in BRI2-Aβ mice.** (**A**) The proportion of the exploration of center of the arena to total exploration path during the open-field test carried out at the age of 14.4 months. (**B**) Latency to fall from the accelerating rotarod performed at the age of 14.6 months. (**C**) Swim paths of 15.1 month-old mice to a visible platform in WM. (**D**) Path tortuosity, expressed as the sum of absolute changes in walking direction divided by total path length. The lower tortuosity of BRI2-Aβ42 mice during first session (S1) did not differentiate the genotypes (F(2,24) = 2.3, p = 0.1), and the tortuosity of these mice did not differ from tortuosity of both BRI2-Aβ40 and non-Tg mice (p = 0.4 and p = 0.15, respectively, *t*-test with Bonferroni adjustment). Error bars represent s.e.m.

In Study 2, we evaluated BRI2-Aβ1-40 and BRI2-Aβ1-42, along with the non-Tg littermates within the age range of 14–17 months, when robust Aβ1-42 pathology is present in the forebrain of BRI2-Aβ1-42 mice [7]. The evaluation commenced at the age of 14.2 months and revealed that both BRI2-Aβ lines and non-Tg mice showed comparable levels of exploration of OF arena and its inner part (Figure 3A, Table 1). Additionally both BRI2-Aβ lines showed comparable to non-Tg littermates latency to initiate exploration, length of exploratory path and its tortuosity (Table 1). The approach and exploration of the object placed in the center of the arena were also similar between tested genotypes (2.7% ± 1.2, 3.2% ± 1.7, 2.0% ± 1.2,% of time exploring object, for non-Tg, BRI2-Aβ1-40, and BRI2-Aβ1-42 mice, respectively, Table 1). Overall males explored the arena less than females (F(1,21) = 5.5, p < 0.05), however, both the ratio of exploration between inner and outer zone and object exploration were comparable between sexes. Also, no significant interaction between sex and genotype was found in the analysis of any of the variables. Locomotor balance and coordination of mice was evaluated in the rotarod test at the age of 14.6 months. There was no significant effect of the genotype (Table 1), and all mice significantly increased their time on the rotating rod during the three sessions F(2,48) = 7.3, p < 0.01, Figure 3B). The main effect of sex and sex by genotype interaction were not significant. Next, the mice were tested in the visible platform version of the WM test at the age of 15.1 months. All mice showed rapid improvement in reaching the cued platform (F(2.42) = 40.8, p < 0.001, RMANOVA), and showed comparable swim paths (Figure 3C, Table 1) and swim speeds (Table 1). Also, all mice improved their orientation to the visible cue, showing less tortuous path during successive trials (F(2,42) = 38.9, p < 0.001, RMANOVA, Figure 3D, Table 1). No significant interactions between the factors were found. The inspection of Figure 3D revealed that BRI-Aβ1-42 mice showed visibly less tortuous path during the first training session. However, the post hoc analysis of tortuosity for the first session revealed no significant differences between the genotypes (F(2,21) = 2.0, p = 0.2), sex (F(1,21) = 0.1, p = 0.8) or genotype by sex interaction (F(2,21) = 0.2, p = 0.8), and none of the pair wise comparisons reached significance at α = 0.05.

**Figure 4:**
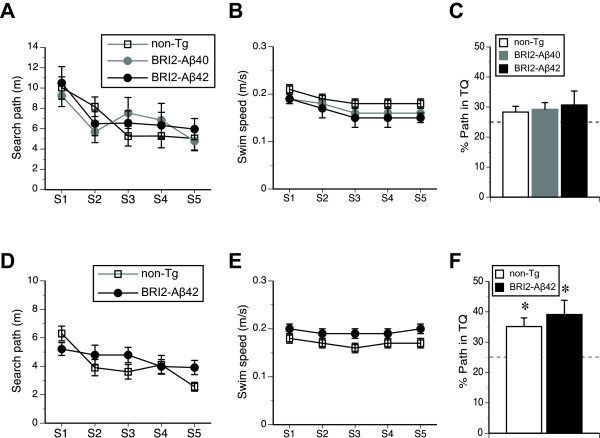
**Normal spatial reference memory in BRI2-Aβ mice.** Spatial reference memory was tested in two cohorts of mice. First cohort (panels **A**-**C**) underwent a battery of non-mnemonic test (Figure 3) and was tested in spatial reference memory WM test at the age of 15.3 months. A separate cohort of experimentally naïve mice (panels **D**-**F**) was tested at the age of 17 months. (**A**) Search path, (**B**) average swim speed and (**C**) search bias for the platform location in the target quadrant (TQ) during the probe trial carried out at the end of training for 15.3 month-old mice. Panels **D**, **E**, and **F** represent search path, swim speed and search of TQ, respectively for naïve 17 month-old mice. S1 - S5 represent daily training sessions. The dashed lines (panels **C** and **F**) represent a chance level performance. Error bars represent s.e.m. * *p* < 0.001 indicates the comparison of each genotype against 25% chance level performance.

**Figure 5:**
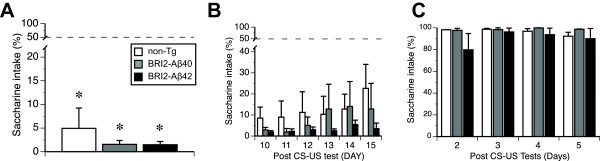
**Normal acquisition and retention of conditioned taste aversion in BRI2-Aβ mice.** Experimentally experienced mice (Figures 3 and 4A-C) were tested at the age of 15.8 months. All genotypes showed strong and resistant to extinction memory of the association between the taste of saccharine and gastric nausea. Conditioned taste aversion was not impaired by selective expression of Aβ1-40 or Aβ1-42 in the brain. (**A**) Saccharine intake in two-choice test carried out on D2 after CS-US pairing. (**B**) Strong and resistant to extinction saccharine avoidance in the series of retention tests carried out between D10 and D15 after CS-US pairing. (**C**) Control (saline injected) unconditioned mice showed strong and stable preference for saccharine. The dashed line (panels A and B) represents a chance level performance. Error bars represent s.e.m. * *p* < 0.001 indicates the comparison of each genotype against 50% chance level performance.

We next evaluated spatial reference memory of the mice in the WM test at 15.3 months of age. The results revealed that non-Tg, BRI2-Aβ1-40, and BRI2-Aβ1-42 mice showed a significant improvement in their search paths for the submerged escape platform during training (F(4,84) = 10.8, *p* < 0.001, RMANOVA, Figure 4A), with no significant effect of the genotype (Table 1), or 2-way and 3-way interactions between the factors. The genotypes did not differ in their swim speed during training (Table 1, Figure 4B), but overall females swam faster than males (F(1,21) = 10.1, p < 0.01), which resulted in their slightly longer, although not significantly different, search paths (7.0 ± 0.7 and 6.7 ± 0.7, for females and males respectively). None of the interactions involving sex was found significant. The mice of all three genotypes demonstrated comparable spatial memory evaluated in a probe trial at the end of training (Table 1, Figure 4C). The memory for the platform was also comparable between sexes (F(1,21) = 2.0, p = 0.17), however, males showed a tendency of stronger memory than females (32.2 ± 3.1 and 27.2 ± 7.2, for males and females respectively, t(25) = 1.4, p = 0.2). The post hoc analysis of the strength of the spatial memory revealed no significantly higher from 25% chance performance memory scores for all 3 genotypes (Figure 4C). In order to eliminate the possibility of floor effect in the spatial memory development, we repeated the WM test using a separate cohort of naïve 17 month-old BRI2-Aβ42 mice and their non-Tg littermates. The results revealed that similarly to the first WM test, both non-Tg and BRI2-Aβ1-42 mice showed a significant improvement in their search path during training (F(4,76) = 5.8, p < 0.001, RMANOVA, Figure 4D), with no significant differences between the genotypes (Table 1) or interaction effects. The BRI2-Aβ1-42 mice tended to swim faster (Figure 4E), however, not significantly (Table 1). Also, spatial memory was comparable between the genotypes (Table 1), and both BRI2-Aβ1-42 and non-Tg controls showed significant bias for the platform location during a probe trial test (t(10) = 2.6, p = 0.026 and t(12) = 2.5, p = 0.027, comparison against 25% chance level for BRI2-Aβ1-42 and non-Tg respectively, Figure 4F). The overall comparison between the two cohorts revealed that mice tested at 15 months had significantly longer search paths during training than naïve 17 month old mice (F(1,35) = 14.1, p < 0.01, Figure 4A and D). Also, females showed longer paths than males (F(1,35) = 4.8, p < 0.05), and faster swim speed than males (F(1,35) = 11.7, p < 0.01). None of the other between subject factors were significant at α = 0.05. The analysis of the spatial memory revealed significantly stronger than 25% chance level memory bias (32.0 ± 3.2, t(22) = 2.2, p < 0.05 and 34.7 ± 3.5, t(19) = 2.8, p < 0.02, for non-Tg and BRI2-Aβ42 mice respectively), with no significant differences between the genotypes and sexes. The CTA test was performed at the age of 15.8 months on mice that were repeatedly tested in the whole behavioral test battery. The results revealed that all genotypes showed strong and comparable memory of the association between novel taste of saccharine and experimentally induced gastric nausea (Table 1), significantly avoiding the taste of saccharine during the choice test carried out on D2 after CS-US pairing (*ps* < 0.001, one-sample Student *t*-test with scores evaluated against 50% chance level, Figure 5A). The developed memory of taste aversion was comparable between the genotypes (Table 1), and was long lasting and resistant to extinction up to 15 days after initial CS-US pairing (Figure 5B). *Post hoc* analysis of the avoidance of saccharine against 50% chance level revealed that mice of all genotypes significantly avoided the taste of saccharine during all retention tests during extinction series (0.05 > *ps* <0.001, one-sample Student *t*-test). Simple effect trend analyses performed on the avoidance of saccharine during extinction tests carried out between D10 and D15 for each genotype yielded non-significant changes in extinction over time (F(2,40) = 2.0, p = 0.16; F(1,30) = 1.0, p = 0.34; F(5,30) = 1.3, p = 0.31 (RMANOVA*s* with Greenhouse-Geisser adjustment of df), for non-Tg, BRI2-Aβ1-40, and BRI2-Aβ1-42 respectively, one-way repeated ANOVA*s*). Male and female mice did not differ in the rate of the extinction of taste aversion. The lack of the differences between the genotypes in the taste aversion was not caused by the differential intake of saccharine solution by mice during conditioning trial (D1) of the test (1.3 ml ± 0.2, 1.7 ml ± 0.2), 1.5 ml ± 0.2, F(2,20) = 1.3, p = 0.30). Also, naïve to saccharine taste and unconditioned mice showed strong and stable preference for the 0.5% saccharine solution (Figure 5C). Overall, there were no significant correlations between Aβ1-42 levels in the BRI2-Aβ1-42 mice forebrain and any measure evaluating non-cognitive or cognitive behavior obtained in the tests (data not shown).

## Discussion

Given the fact that behavioral impairment is seen in most APP transgenic mouse models [1,27] and after direct injection of synthetic Aβ1-42 or Aβ1-40 into the rodent brain [28-31], the current findings in BRI2-Aβ mice are novel and unexpected. Our present results showed conclusively that neither BRI2-Aβ line showed compromised fear conditioned memory at the stage of early onset of Aβ pathology, which is characterized by a significant increase in relevant soluble Aβ in single transgenic BRI2-Aβ lines, or by an increase in both Aβ1-42 and Aβ1-40 peptides in double transgenic mice [24]. These results are apparently in contrast with the evaluations of APP transgenic mice in fear conditioning test, which demonstrated clear memory impairments in Tg2576 model at ages preceding Aβ deposition [32]. In our previous studies, we also demonstrated age-dependent decline of conditioned fear memory in APP CRND8 mice [25]. Our results also indicate that the presence of aggregated extracellular Aβ42 species along with concurrent overt Aβ42 amyloid plaque deposition at later ages did not affect motivation to explore novel environment of the open-field arena, their motor coordination, or swimming ability. To strengthen experimental and external validity of our study and to eliminate potential false negative results of cognitive evaluation, which could be observed only in one test or even limited to specific experimental settings [33], we characterized multiple memory systems of BRI2-Aβ mice. The chosen experimental paradigms were successfully used in our lab to demonstrate impairment in APP CRND8 mice, and focused on spatial learning and reference memory evaluated in WM test [9,10,13] and conditioned taste aversion, a form of Pavlovian associative learning, [34], in addition to the context and tone conditioned fear memory paradigm [25], used with younger cohorts of BRI2-Aβ mice. Overall, our results suggest that chronic exposure to aggregated extracellular Aβ is not, by itself, sufficient to cause memory impairment in mice. This general conclusion is further substantiated by the fact that the effect size or amount of variance explained by the genotype was either negligible to low (ranging from 0 to 0.09, Table 1). This discrepancy in cognitive profiles between BRI2-Aβ and APP transgenic mice, for example APP CRND8 model tested intensively in our laboratory, cannot be easily attributed to the mouse genetic background, which is known to modulate behavior [35-37], since genetic background is comparable between BRI2-Aβ and CRND8 models [7,9]. In addition, interaction with testing environment [38,39], or personnel cannot explain the discrepancy, since the cognitive tests in the present study were run by the same PI (CJ) and assistant (AH) as our previously published work on APP CRND8 mice [10,25,34].

The inclusion of males and females in our study was driven by the hypothesis that the cognitive characterization of both sexes in the study might provide more sensitive scenario of modeling human Alzheimer’s disease in which prevalence in the time of onset and/or increased severity in women were reported [40-42]. Also, the increase in amyloid load in females was also reported in some APP transgenic mouse models [43,44]. Although, our results did not reveal any significant differences behavioral between males and females within each studied genotype, which further substantiates the overall lack of the effect of increased levels of Aβ1-42 on behavior, we observed overall sex effect in tests known to differentiate the behavior of sex in mice. Recognizing the fact that sex differences are variable and dependent on the strain of mice [45], we observed increased exploratory activity of the OF arena by females, confirming previously published reports [46,47], and inferior performance of females in the WM test, also reported previously [48-51].

The present results raise a number of substantive questions regarding behavioral deficits in APP overexpressing transgenic mice. Primarily, they point to possible roles of full length APP or other APP derivatives in mediating Aβ-associated toxicity in mouse models. This hypothesis is supported by recent evidence demonstrating that conditional suppression of mutated human *APP* transgene at the stage of florid Aβ pathology in a mouse model restored learning propensity of the mice, despite the presence of abundant Aβ plaques in the brain [52]. Another possibility is that seemingly toxic Aβ oligomers may not be homogenous assemblies of Aβ, but may contain other APP derivatives that contribute to toxicity [53]. Alternatively, other APP fragments may mediate toxicity. The accumulation of β-secretase cleaved fragments of APP has been implicated as mediators of behavioral dysfunction, and previous studies have shown that aggregated Aβ can interact with APP [54-56]). Although overall amyloid plaque pathology was comparable between 17 month old BRI2- Aβ1-42 mice and 4 month old APP CRND8 mice (Figure 1E, 1F), BRI2- Aβ1-42 mice have Aβ42 amyloids, lacking Aβ40 or other derivatives. Therefore, we cannot exclude the possibility that Aβ40 or other derivatives are required to trigger cognitive deficits in mice. In future study, it will be informative to test cognitive performance with very old BRI2-Aβ1-40/ BRI2-Aβ1-42 mice that have extensive amyloid deposition. However, extensive premature death phenotype in the bitransgenic mice has precluded rigorous evaluation of cognition at old age.

The notion that Aβ aggregates are not sufficient to induce memory dysfunction in mice does not contradict the results of the studies that show that synthetic Aβ or Aβ aggregates injected into rodent brain may mediate memory formation e.g. [28,57]. Our current findings would parsimoniously suggest that such acute interventions in the adult brain might be more toxic, and might not reflect the effects of chronic exposures to increasing levels of extracellular soluble or aggregated Aβ in the brain during development, a process which models AD-like amyloid formation in more realistic developmental time frames. In our models, the prolonged and more physiological secretion of Aβ with progressing Aβ aggregation and accumulation seem to replicate more realistically the progression of Aβ pathology in the brain during ontogeny and the neuronal response to Aβ lesions.

Interestingly, it has been reported that selective expression of Aβ1-42 and Aβ1-40 in the brain using virally-mediated gene transfer of BRI2-Aβ fusion proteins modified behavior in a rat model [58]. However, the reported behavioral outcomes were not fully consistent with memory impairment. Overall, except for reported hyperactivity in the open-field test in rats over-expressing both BRI2-Aβ1-42 and BRI2-Aβ1-40 peptides, the AAV-BRI2-Aβ injected rats, especially the cohort injected with BRI2-Aβ1-42, showed comparable spatial learning and memory, short-term memory of object recognition, and the latency to re-enter a chamber associated with foot-shock in a passive avoidance test to control-injected rats. The apparent lack of a consistent trend in behavioral changes across behavioral tests in AAV-BRI2-Aβ1-42 rats and apparently their unimpaired spatial and short-term object recognition memories [58] are consistent with the lack of cognitive impairment in BRI2-Aβ1-42 mice observed in the present study.

## Conclusions

Overall, the observed dissociation between Aβ accumulation in the brain of BRI2-Aβ1-42 mice and cognitive decline provides a novel research tool to investigate the formation of molecular assemblies and conformational changes, as well as contribution of additional factors which may lead to amyloid-β toxicity and consequently to memory decline [59].

## Methods

### Mice

BRI2-Aβ mice, maintained on hybrid C57/B6//C3H background, were generated as described previously [7]. All mice were hemizygous for the respective BRI2-Aβ transgenes. To generate BRI2-Aβ1-40/Aβ1-42 mice, BRI2-Aβ1-40 and BRI2-Aβ1-42 mice were crossed together. Non-transgenic littermates served as controls in all experiments. Mice were habituated to the experimental handling one week before the onset of experiments. They were removed from a home cage using hand-cupping method [60] and their body weight was recorded on the last day of pre-experimental handling. We used cohorts of mice bred in two different animal facilities. Two cohorts were obtained from the breeding stock at the University of Florida, Gainesville; cohort 1, comprised of 72 mice (23(12m:11f), 18(10m:8f), 19(9m:10f), 12(5m:7f) for non-Tg, BRI2-Aβ1-40, BRI2-Aβ1-42, and BRI2-Aβ1-40/Aβ1-42 respectively, m - males; f – females) at the age of 12 months, was tested in fear conditioning paradigm, and cohort 2, of 24 mice (12(5m:7f) and 12(6m:6f) for non-Tg and BRI2-Aβ1-42 respectively) at the age of 17 months, was tested in the spatial reference memory of the water maze test. A cohort of 27 mice at the age of 14 months (10(4m:6f), 8(4m:4f), 9(4m:5f) for non-Tg, BRI2-Aβ1-40, BRI2-Aβ1-42 respectively) at the onset of the experiments was obtained from Mayo Clinic, Jacksonville, FL, breeding stock, and was subjected to a battery of behavioral tests including: open-field (OF), rotarod (RR), visible platform (VP) and spatial reference (SR) memory versions of the water maze tests, and conditioned taste aversion (CTA) test. The tests were administered following longitudinal experimental design at ages of 14.2, 14.6, 15.1, 15.3, and 15.8 months respectively. One male in BRI2-Aβ1-42 group died before the onset of CTA test, reducing the sample size of this group to 8 mice. The tests in the battery were administered in the above listed order, from least to most invasive [61], to minimize affecting behavior by prior test history. Institutional Animal Care and Use Committees of University of Florida and Mayo Clinic approved all experiments.

### Locomotor activity in open-field (OF) test

The exploration of a novel environment was evaluated in an open-field arena as described [12]. The white OF arena was 120 cm in diameter, surrounded by a 40 cm wall. Overhead incandescent light provided illumination of about 700 lx in the arena. A cylindrical (5 × 10H cm) object was placed in the center. Each mouse was tested in 3 consecutive daily 5-min sessions, with 3–4 min interval in between sessions. A mouse exploratory behavior was recorded by an image tracking system (HVS Image, Buckingham, UK). The following measures were extracted using Wintrack program [62]: latency to start exploration, walking path (m) and walking speed (m/s), % path in the wall zone (30 cm), path tortuosity (˚/m), time spent immobile, object approach and total time and frequency of object exploration. The data were averaged across the 3 sessions for the analysis.

### Motor coordination in rotarod (RR) test

The mice were trained in squads of 4 in the Rotamex-5 apparatus (Columbus Inst. OH). One day before the test the mice were given 3, 5-min pre-training trials with constant (5 rpm) rotation of the rod. Following, mice were tested in 3 consecutive days, with 3, 5-min trials (inter-trial-interval of 40–50 min) and gradual acceleration of the rod from 4 to 40 rpm within 5-min trial. The speed and the latency to fall from the accelerating rod were recorded. In the case of passive rotation (a mouse loses its balance, but clings to the rod and recovers balance after a full rotation), the latency to the first complete passive revolution was recorded.

### Fear conditioning (FC) test

The test was performed as previously described [25]. A 4-chamber conditioning apparatus (Coulbourn Inst.) was located in a dedicated room. A tone (80 dB, pulse (6 clicks per second), 30-s duration) was used as conditioned stimulus (CS) and a 0.45 mA, 2s foot shock, which co-terminated with a tone, as unconditioned stimulus (US). Mouse activity was recorded by FreezeFrame (Actimetrics) program, and freezing behavior, defined as cessation of all movements other than respiratory activity [63], indicating fear memory of the association between CS and US was analyzed off-line. Each mouse received 2 CS-US pairings separated by a 60-s interval during one 5-min training session. After a day of recovery (D3), the fear-induced freezing of mice was evaluated in the context of the original training chamber (contextual fear memory), and a day later (D4) the fear memory elicited by tone only was evaluated in a test carried out in the modified context of the chamber (tone fear memory). Both tests were carried out in an extinction mode with no shock administered.

### Water maze (WM) test

Mice were trained in the water maze test as described [64]. The *reference memory version* of the test was run for 5 consecutive days with 4 60-second training trials per day. A mouse was released into water at semi-randomly chosen cardinal compass points (N, E, S, W [11]) and its swim path was recorded by image-tracking software (HVS Image). Dark, geometrical shapes (2–3 per wall), a cabinet, and divider curtains, separating an experimenter from the testing area, served as spatial cues in the room. An escape platform, submerged 0.5 cm under water surface, was positioned in the center of the same quadrant of the pool (target quadrant, TQ) throughout training. The memory for platform location was evaluated in a probe trial (with escape platform removed) 24 h after the last training trial. During a *visible platform* test, run for 3 days with 4 trails per day during a week preceding spatial reference memory training, the platform was marked by a visible black cue and a curtain surrounded the pool.

### Conditioned Taste Aversion (CTA) test

The CTA test, which evaluates the association of the novel taste (CS) with experimentally induced nausea (US), was carried out as described [34]. On the day of conditioning, mice, deprived of water over night, were allowed to drink 0.5% saccharine (2,3-Dihydro-3-oxobenzisosulfonazole, SIGMA) solution (CS) provided in a 15 ml bottle during a 30-min morning session. One hour later, the conditioned group was injected intra-peritoneally with lithium chloride (LiCl; 0.14M, 2% body weight) as a nausea-inducing agent (US), while the control group was injected with corresponding amount of saline. A day later (D2), overnight water deprived mice were given a two-bottle choice test between water and saccharin solution. Placement of saccharine bottles with reference to the water bottles during the test was random, following the method used in our previously published study [34]. The choice test was repeated daily from D10 to D15 after the CS-US pairing in order to determine the rate of memory extinction. Saccharine preference index was expressed as the percent of saccharine intake to total fluid intake (ml saccharine/(ml water + ml saccharine) * 100).

### Histochemical staining

Paraffin-embedded tissue was sectioned at a thickness of 5μm and mounted on glass slides. After removal of paraffin in xylene and rehydration in a series of alcohol solutions (100% and 95%), sections were steamed for 30 min in distilled water for antigen retrieval. Immunostaining was performed with anti-total Aβ antibody (33.1.1, 1:1,000; [65]) on a DAKO autostainer according to the manufacturer’s instructions using DAKO envision1 system peroxidase kit, with 3,3-diaminobenzidine as the chromogen (DAB; Dako, Carpinteria, CA). After immunostaining, sections were counterstained with hematoxylin and coverslipped.

### Aβ sandwich ELISA

Forebrains were homogenized in radioimmunoprecipitation assay (RIPA) buffered with 1× protease inhibitor cocktail (Roche). The homogenate was ultracentrifuged at 100,000 × g for 1 h at 4°C to collect RIPA-soluble fractions. RIPA-insoluble pellets were sonicated in 2% SDS, ultracentrifuged at 100,000 × g for 1 h at 4°C to collect SDS-soluble fractions. Lastly, the SDS-insoluble pellets were sonicated in 70% formic acid (FA) and ultracentrifuged at 100,000 × g for 1 h at 4°C to yield the formic acid fraction. The following dilutions of the brain lysates were used in Aβ ELISAs: For 12 mo Bri42: RIPA 1:10, SDS 1:50, FA 1:00; For 17 mo Bri42 and 4 mo CRND8: RIPA 1:10, SDS 1:60, FA 1:300.

Aβ40 levels were determined by Aβ sandwich ELISAs using Ab9 (anti-Aβ1-16) as the capture antibody and 13.1.1-HRP (anti-Aβ35-40) [65] as the detection antibody for Aβ1-40. Aβ1-42 levels were measured by using Ab2.1.3 (anti-Aβ35-42) [65] as the capture antibody for Aβ1-42 and Ab9-HRP as the detection antibody.

### Aβ Western blotting

SDS brain lysates, heated at 50°C for three minutes in the presence of denaturing sample buffer, were separated on 16.5% Tris-Tricine gel (Bio-Rad) in 1x Tris/Tricine/SDS running buffer. The SDS-PAGE resolved samples were transferred 0.2 μm nitrocellulose membrane, boiled for 5 minutes in TBS, blocked in Starting Block (Thermo Scientific, Waltham, MA) and incubated overnight in 82E1 antibody (Aβ1-16; IBL, Hamburg, Germany). Detection was performed with donkey anti-mouse antibody conjugated to HRP (Jackson ImmunoResearch, West Grove, PA). Chemiluminescence signal was visualized using West Femto Chemiluminescent Substrate (Thermo Scientific) with a FujiFilm system.

### Statistical analysis

Factorial analysis of variance (ANOVA), with genotype and sex as between subject and sessions as repeated factors (RMANOVA), was used to analyze data. Omega squared (ω^2^) is reported as the estimate of the effect size accounted by the genotype of mice to augment significance of statistical tests. Omega squared estimates the proportion of systematic variance in the population instead of the sample [66], thus allowing better generalization of results, and it is relatively independent of sample size [67,68]. Simple effects were evaluated using one-way ANOVA. When necessary, degrees of freedom were adjusted by Greenhouse-Geisser epsilon correction for the heterogeneity of variance. In multiple planned comparisons, the Bonferroni adjustment of α level minimizing Type I error rate was used. Comparisons between two independent groups were done using a Student *t*-test. Comparisons against chance performance (25% in the case of search path in the TQ; and 50% in the case of saccharine intake in CTA) were done using one-sample *t*-test. Spearman’s rank correlation was used to assess the associations between Aβ levels and behavior. The critical α level was set to 0.05. Only significant results are reported in a text, while all ANOVA*s* results pertaining to the genotype effect are reported in Table 1.

## Abbreviations

Aβ: Amyloid β; Ab: Antibody; ABRI: Amyloid BRI; APP: Amyloid precursor protein; AD: Alzheimer’s disease; ANOVA: Analysis of variance; BRI: British, referred to gene, protein, etc.; β-CTF: β-carboxyl-terminal fragments; CRND8: Transgenic mouse model of AD-like amyloidosis; CS: Conditioned stimulus; CTA: Conditioned taste aversion; D: Day; ELISA: Enzyme-linked immunosorbent assay; kD: Kilo Dalton; LiCl: Lithium chloride; LTP: Long-term potentiation; NMDA receptor: *N*-methyl-D-aspartate receptor; non-Tg: Non-transgenic; OF: Open field; PS1: Presenilin 1; RIPA: Radioimmunoprecipitation assay buffer; RMANOVA: Repeated measures ANOVA; RR: Rota-rod; sAPPβ: Amino-terminal-soluble APPβ; SR: Spatial reference; Tg: Transgenic; Tg2576: Transgenic mouse model of AD-like amyloidosis; TQ: Target quadrant; US: Unconditioned stimulus; VP: Visible platform; WM: Water maze.

## Competing interests

The authors declare no competing financial interests regarding this work.

## Authors’ contributions

JK, PC, AH, and AM jointly performed research. DD assisted in research (brain pathology analyses). CJ and JK designed research, and CJ analyzed data. CJ, DRB, TG, and JK wrote the manuscript. All authors read and approved the final manuscript.
